# Spin dynamics investigations of multifunctional ambient scalable Fe_3_O_4_ surface decorated ZnO magnetic nanocomposite using FMR

**DOI:** 10.1038/s41598-021-83394-8

**Published:** 2021-02-15

**Authors:** Saurabh Pathak, Rajni Verma, Sakshi Singhal, Raghav Chaturvedi, Prashant Kumar, Pragati Sharma, R. P. Pant, Xu Wang

**Affiliations:** 1 School of Engineering, RMIT University, Melbourne, VIC Australia; 2 Department of Mechanical Engineering, The University of Melbourne, Parkville, VIC 3010 Australia; 3 Academy of Scientific and Innovative Research, CSIR-NPL Campus, New Delhi, India; 4 School of Physics, The University of Melbourne, Parkville, VIC Australia; 5 Institute of Nuclear Medicine & Allied Sciences, DRDO, Brig SK Mazumdar Road, Delhi, India; 6 Technische Universität Dresden, Dresden, Germany

**Keywords:** Chemistry, Engineering, Materials science, Nanoscience and technology

## Abstract

Microwave spin resonance behavior of the Fe_3_O_4_ surface decorated ZnO nanocomposites (FZNC) has been investigated by ferromagnetic resonance (FMR). Modified hydrothermal method has been adopted to fabricate FZNC samples with Fe_3_O_4_ nanoparticles chains were used as seeds in the uniform magnetic field to decorate them on the surface of the ZnO nanoparticles in a unique configuration. Spin dynamics investigation confirms the transition of ZnO from diamagnetic to ferromagnetic as the sharp FMR spectra converts to the broad spectra with Fe_3_O_4_ nanoparticles incorporation. A single broad FMR spectra confirms that no isolated Fe^3+^ or Zn^2+^ ions exist which is also in agreement with XRD confirming suitable composite formation. Further, the increase in Fe_3_O_4_ concentration leads to decrease in g-value which is resulting from the internal field enhancement due to magnetic ordering. Also, various spin resonance parameters were calculated for the FZNC which provides a detail information about the magnetic ordering, exchange coupling and anisotropy. Elemental analysis confirms the presence of Fe and Zn simultaneously and transmission electron microscopy (TEM) image show the presence of Fe_3_O_4_ on the grain boundaries of ZnO which has been confirmed by taking high-resolution TEM and electron diffraction patterns on both sides of the interface. These unique structural configuration of the FZNC has tremendous potential in various magneto-optoelectronic, spintronics and electro-chemical applications.

## Introduction

Magnetic manipulation in materials achieved through hybrid materials are of great interest owing to their unique physical, chemical and optical properties which can be tuned by external magnetic field^1^. Magnetic control on the materials properties has been an exciting area of research as they exhibit immense potentials for the device development with enhanced performance^2^. One of the most vital part of both basic research and applications of these composites lies in rational understanding the spin dynamics of these materials to achieve the desired properties^3^. Metal oxide materials are widely used in engineering as well as biomedical applications. In particular, zinc oxide (ZnO) of wurtzite hexagonal structure (space group- C_6v_^4^-P6_3_mc, direct band gap 3.37 eV) is one of the most prominent materials with wide interdisciplinary applications in optoelectronic, photo-catalysis, sensing, and drug delivery^4,5^. However, nanotechnology has opened a whole new horizon of several biomedical application such as bio-imaging/sensing, anti-microbial/cancer and drug/gene delivery etc. as nanostructures behaves in contradicting manner from their bulk counterparts due to enhanced surface dependent properties and large defect sites^6^.

Magnetic nanocomposites (MNC) or magneto-hybrid materials are multiphase materials with one or more magnetic phases and one of the phases present in the nanoscale range^7^. Composite materials generally show properties that are inherently different from the present phases or encompasses a combination of the property of all the present phases in the system. The MNCs are solid solutions of two or more components in which the matrix of one material is mixed with the other material to obtain the desired properties. Superparamagnetic (SP) Fe_3_O_4_ particles of inverse spinel AB_2_O_4_ structure with A (octahedral) site occupied by Fe^2+^ ions and B (tetrahedral) sites occupied by Fe^3+^ ions has high magnetic saturation (M_s_ = 40 emu/g with average particle diameter = 10 nm) which makes them effective as a hybrid material^2,8^. SP Fe_3_O_4_ nanoparticles are ideal for their use in combination with ZnO as it has a narrow bandgap, biocompatible, non-toxic, high chemical stability, mechanical hardness, and high saturation magnetization (M_s_) material^9^.

The embodiment of the magnetic nanoparticles in ZnO provides a unique blend of properties as ZnO provides excellent optoelectronic properties and incorporation of magnetic nanoparticles provides an opportunity to tune these properties through an external magnetic field. The unique magneto-optoelectronic properties of these materials have engrossed the research focus on the synthesis of Fe_3_O_4_-ZnO nanomaterials (FZNM) in various morphologies and structures. Also, FZNM fabrication has been reported in wide domain and interdisciplinary field such as biomedical, optoelectronics, electrochemical, and photocatalytic due to its enhanced magnetic field induced magneto-optoelectronic, spintronic, and electrochemical performance. Altogether, heterostructure FZNM has tremendous potential to be efficiently used in spintronic devices. The ferromagnetic alignment in ZnO by incorporating magnetically active material provides new landscapes in the field of magneto-optoelectronic, spintronics, and electrochemical devices^10^.

The existence of the strong correlation between the structure of the composite to its physical and chemical properties requires exploration of different nanocomposite structures and its optimization. Major attention in the fabrication of the FZNM is required to attain isotropic properties by uniformly distributing the nanoparticles in the matrix. Matrix dispersed structures are useful when uniformity of the properties is required throughout the material. The molten salt method is one pot novel technique for the synthesis of matrix dispersed magnetite-zinc oxide nanocomposites. Reddy et al.^5^ prepared Fe_3_O_4_-ZnO hybrid nanocomposite for application in anode material in Li-Ion batteries. They have prepared the composite by the molten salt method in the average size range above 100 nm. ZnO acts as a good matrix element for Fe_3_O_4_ imparting it exceptional Li-ion recycling properties. The two-step approach adopted by S. Singh et al^11^ for the fabrication of Fe_3_O_4_ embedded ZnO magnetic semiconductor nanocomposites has shown great potential and has achieved good isotropic properties. In the first step, glycine functionalized magnetic nanoparticles were prepared which are mixed with the Zn precursors and refluxed to in-situ fabricate ZnO. The prepared composite displays remarkable detoxification properties which were used for the removal of bacterial pathogens.

Further, core–shell structures are highly efficient as they provide excellent stability, dispersibility, and functionality to the composite materials. Constituents of the core–shell and their ratio can be manipulated to achieve the desired properties required by the application. Seed mediated growth process and sequential nanoemulsion techniques have been successfully employed for the synthesis of core–shell nanostructures ^4,12,13^. Jian Wang et al^14^ prepared Fe_3_O_4_-ZnO core–shell nanocomposites by a simple two-step chemical method of size range 60 nm for their use in wastewater treatment. They have not observed any degradation of photocatalytic properties after several cycles of water treatment suggesting a high performance of the material. Magnetic properties imparted by Fe_3_O_4_ core ensured the re-usability of the composite. These core/shell nanocomposites were employed in the fabrication of inverted solar cells for their enhanced performance. The incorporation of magnetite nanoparticles leads to an increase in short circuit current density due to the presence of local magnetic field and ZnO nanoparticles suppressed parasitic absorption of radiation by magnetic nanoparticle^15^. Further, multicore-shell structures are sculptured when a magnetic layer is needed to be sandwiched between two non-magnetic layers. Interfacial interactions at the two surfaces of the core nanoparticles impart unique properties to the material. Multifunctionality of the structure facilitates their use as a probe for target-specific imaging and drug delivery agents^16^. Uniform spatial distribution of properties necessitates the fabrication of Janus structures Fe_3_O_4_-TiO_2_ nanocomposites prepared by Zeng et al^17^ shows good imaging properties and can be employed in photodynamic therapies.

Probing the spin dynamics of the nanocomposite can provide us with new insight enabling the improvements in the functionality of the composite. Understanding the dipolar and superexchange interactions existing among the particles is essential to comprehend the dynamics of the composite^18^. Spin resonance studies of pure ZnO and Fe_3_O_4_ nanoparticles using the FMR technique have been carried out extensively in the past^19,20^. ZnO nanoparticles are diamagnetic but reveal their paramagnetic comportment in the FMR spectrum. The peak observed in the FMR spectra for ZnO stipulates the existence of paramagnetism^21^. Further, the presence of oxygen vacancies and interstitial zinc ions are responsible for the manifestation of paramagnetic behavior in the resonance spectra. The random orientation of electron spins in ZnO realigns itself altogether into the direction of the applied magnetic field absorbing energy in a narrow band of frequencies. Due to the simultaneous realignment of all the spins, a narrow linewidth is observed in the ZnO FMR spectra. On the other hand, Fe_3_O_4_ nanoparticles are SP in nature and exhibit high spin polarization at room temperature^22^. Anisotropy energy enforces the magnetization direction of individual spins to freeze in a specified direction. Exchange interactions existing among the spins preserves the parallel alignment of the magnetic moments. The cumulative effect of the two interactions results in a parallel alignment of spins in different directions. Due to different directions of spins in neighboring crystallites, absorption of radiation occurs over a wider range of frequencies leading to linewidth broadening. FZNM provide opportunities of resonance enabling widespread applications and components concentration or amount of the constituents in the composite can be optimized to get the resonance properties in accordance with the application implementation^18^.

Numerous reports are available in the literature investigating the structural, magnetic, and electronic properties of FZNC. However, no significant attempts have been made to explore the spin dynamics of FZNC to the best of our knowledge. All the previous FZNC reported in the literature has Fe_3_O_4_ nanoparticles in the core and ZnO in the shell, whereas in the present work we have capitalized the chain structure formation of the Fe_3_O_4_ nanoparticles to occupy the shell of the ZnO core. This configuration has not been reported yet and has several advantages. An intensive literature search has been made and we have not observed any investigation targeting the spin dynamics of FZNC in detail. The understanding of the spin dynamics of the magneto-nanocomposite can open new avenues in the field of magnetic field tunable semiconductor oxides^23^.

Herein we have performed a detailed investigation of the dynamic magnetic properties of multifunctional FZNC. We have adopted a simple facile chemical synthesis technique for the fabrication of the nanocomposite in which Fe_3_O_4_ nanoparticles prepared by co-precipitation technique were used as seeds followed by in-situ hydrothermal growth of the ZnO. The ZnO precursors were mixed with the Fe_3_O_4_ seeds in the presence of the constant magnetic applied radially to assist in chain formation over the in-situ seeding ZnO nanoparticles. This allows Fe_3_O_4_ nanoparticles to coat the ZnO nanoparticles rather than taking the core position. Further, the structural behavior of the prepared nanocomposite has been obtained by the X-ray diffraction (XRD) technique. The obtained XRD pattern for each sample was fitted by Williamson-Hall (W–H) method to calculate the crystallite size and strain. The morphology and size distribution was confirmed by scanning electron microscope (SEM) and transmission electron microscope (TEM). Further, an energy dispersive X-ray spectrum (EDS) was used to obtain the elemental mapping to confirm the structure of the FZNC. The static magnetic properties of the pure ZnO, Fe_3_O_4,_ and FZNC were obtained by vibrating sample magnetometer (VSM) to observe the effect of magnetic nanoparticles incorporation. The spin dynamics of the FZNC was investigated by the microwave spin resonance technique by fitting the FMR spectra of the samples. The FMR spectra obtained for the samples were fitted with various theoretical models presented to establish a relationship among different spin resonance properties. Various spin resonance properties were calculated for each sample and optimized concentrations were obtained, which is the key for efficient material performance.

## Synthesis of MNC samples

**Figure 1:**
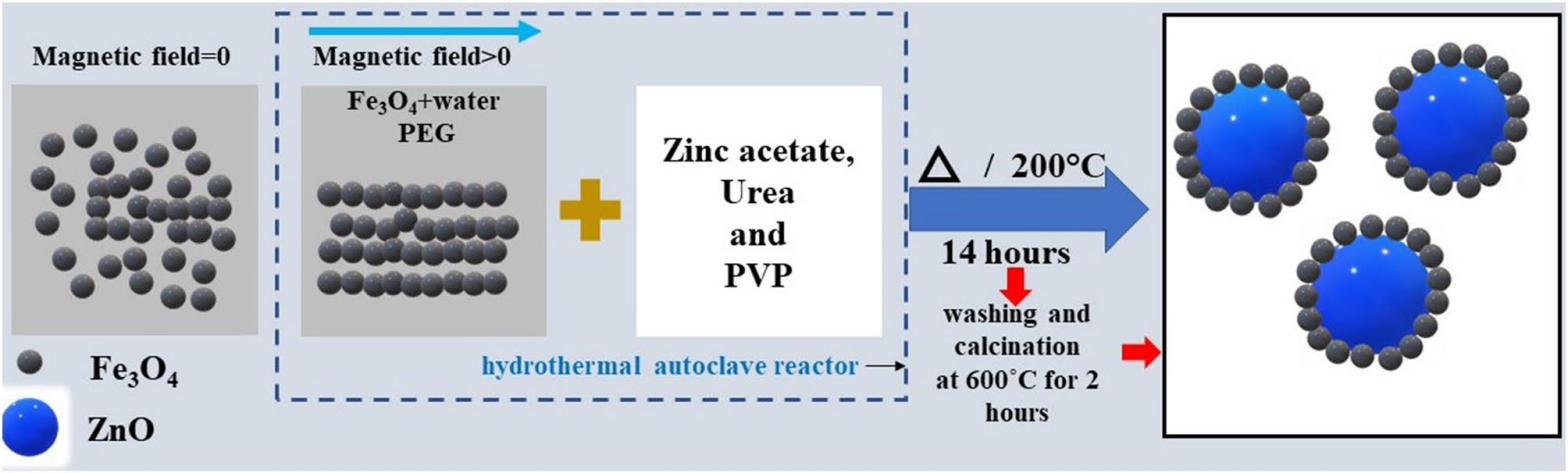
Schematic of the modified hydrothermal process adopted for the synthesis of the Fe_3_O_4_ surface decorated ZnO nanocomposite. Fe_3_O_4_ nanochains were used as the seed to ZnO particles.

A detailed experimental investigation has been performed to investigate the spin dynamics of FZNC to understand their microwave absorption behavior. The schematic of the synthesis of the Fe_3_O_4_ surface decorated ZnO magnetic nanocomposite is depicted in Fig. 1. Multifunctional MNC was prepared in a two-steps process; in the first step, seeds of Fe_3_O_4_ nanocrystals have been prepared by the standard co-precipitation method. Synthesis and surface functionalization of Fe_3_O_4_ nanocrystals were performed by adopting a procedure described before^24^. Further, in the second step, these prepared Fe_3_O_4_ nanocrystals were utilized as seeds for the synthesis of MNC using the hydrothermal method. In the typical procedure, 2 g polyethylene glycol was added in 10 ml of deionized (DI) water at a uniform stirring rate of 400 rpm for 15 min. Dried Fe_3_O_4_ nanocrystals were then added in three different concentrations (25, 50, and 100 mg) under controlled sonication during the mixing process. These three samples were named FZA, FZB, and FZC respectively with increasing Fe_3_O_4_ concentration in the composite system. Afterward, 0.5 M 10 ml zinc acetate solution was added to the above solution dropwise under a constant stirring rate (400 rpm) for 30 min. 10 mL 1 M urea and 0.5 g polyvinylpyrrolidone (PVP), were mixed separately and added to the resultant solution dropwise while maintaining a constant temperature of the solution. The resultant solution was kept in a uniform radial magnetic field with mechanical stirring for 15 min to allow chain formation of the Fe_3_O_4_ nanoparticles providing the shell to grow the ZnO nanoparticles in the core as shown in Fig. 1. The complete solution was transferred into the 100 mL hydrothermal autoclave reactor and kept inside a hot air oven at temperature 200 °C for 14 h. The resultant product was then washed several times with ethanol and DI water and dried in a vacuum oven overnight. The obtained powder was calcined at 600 °C for 2 h in a muffle furnace (CARBOLITE GERO-temperature stability + 2.3 °C)^9,25^. Further uncapped ZnO was prepared by adopting the same procedure and experimental conditions except for the addition of Fe_3_O_4_ nanocrystal. Also, a small amount of Fe_3_O_4_ nanocrystal was taken out for different characterizations in the first step and it has also been probed for all the characterizations to provide an intensive comparison of bare ZnO, Fe_3_O_4_ nanocrystals, and MNC. The details of the samples and nomenclature are shown in Table 1.

**Table 1:** Structural, magnetic and spin resonance parameters of ZN, FZNC and FN samples.

Sample Name	Fe_3_O_4_ content (g)	Crystallite Size (nm)	(M_s_) (emu/g)	$H_{R}$(G)	g-value	$\Delta H_{PP}$(G)	$\Delta H_{1/2}$(G)	$\Delta H_{1/2}$/$\Delta H_{PP}$	N_s_ (10^22^)	T_s_ (10^–10^) s
FN	–	9.30	36.93	3338.7	2.0800	1102.5	1271.3	1.153	261.09	2.702
FZA	0.025	42.40	0.28	3473.0	2.0005	1141.4	575.1	1.634	70.66	6.220
FZB	0.050	41.70	0.56	3260.1	2.1310	1141.8	1286.0	1.126	148.34	2.607
FZC	0.100	44.70	5.70	3201.6	2.1701	1047.8	1299.8	1.240	142.73	2.533
ZN	–	45.03		3526.8	1.9690	6.9981	–	–	–	–

## Results and discussions

**Figure 2:**
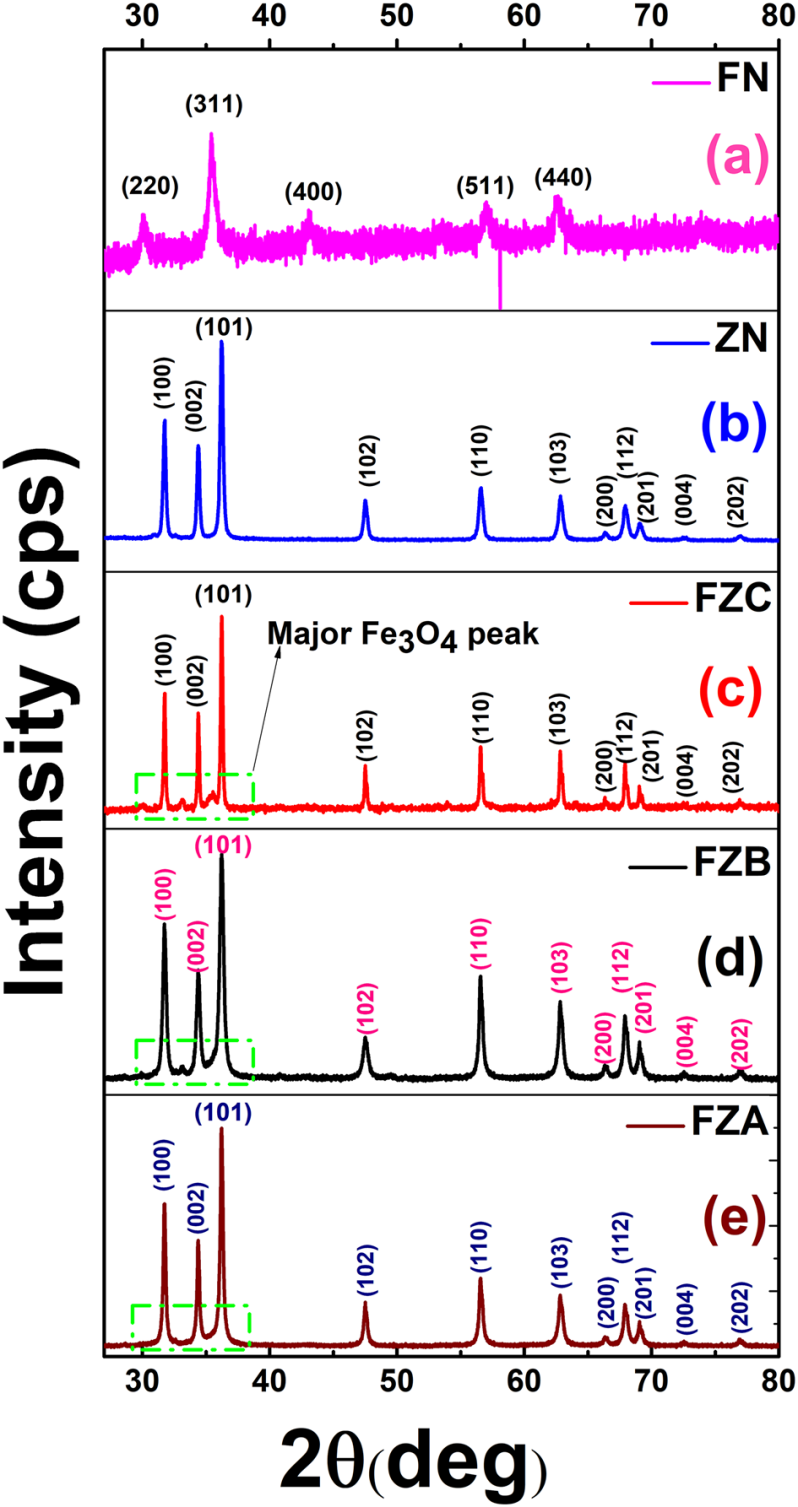
XRD pattern of the FZNC samples (**a**) Fe_3_O_4_, (**b**) bare ZnO, (**c**) FZC, (**d**) FZB, (**e**) FZA.

The XRD plots of FZNC, ZN, and FN samples are shown in Fig. 2(a–e). The XRD pattern of FN is shown in Fig. 2(a) and diffraction peaks perfectly match with the spinel ferrite phase of Fe_3_O_4_ (JCPDS 65–3107) and no extra peaks have been observed which confirms the crystalline purity of the sample. Figure 2(b) shows the XRD pattern of ZN samples and all the peaks of the patterns are in complete agreement with hexagonal ZnO (JCPDS 36–1451). Now, Fig. 2(c–e), shows the XRD pattern of the FZNC samples. Figure 2(d–e) shows the diffraction patterns of the samples FZB and FZA, respectively, in which the peaks are in accordance with the hexagonal ZnO with no separate peak of the Fe_3_O_4_ cubic crystalline phase. However, a small variation in the diffraction pattern can be seen at peak 35.4° in FZA and FZB samples at the major peak of Fe_3_O_4_ cubic phase as this peak is completely merging with the ZnO peaks positions of 34° and 36°. The deviation at 35.4° is evident in the presence of Fe_3_O_4_ cubic phase and its complete merger confirms the formation of excellent composite with uniform completely mixed in ZnO. Also, the peak does not appear abruptly which signifies that multiphase composite will perform efficiently as composite due to effective incorporation of Fe_3_O_4_ in ZnO^26^. However, in the XRD pattern of FZC (Fig. 2(c)) the small peak appears abruptly at 35.4°, which corresponds to Fe_3_O_4_ major peak (311) and indicates that Fe_3_O_4_ is not properly mixed with the ZnO in FZC and appears separately in the nanocomposite. From this we can understand that the use of optimized concentration of Fe_3_O_4_ is desired for efficient hybrid material. The crystallite sizes obtained from W–H plots are 9.3 nm (Fe_3_O_4_), 45 nm (bare ZnO), 42.4 nm (FZA), 41.7 nm (FZB), and 44.7 nm (FZC)^27^. From the XRD patterns, we have observed that the optimized concentration of FN in nanocomposite does not affect the structural properties significantly and appears as a separate phase. It is not mixed with the ZnO matrix which signifies that the composite will show properties of both of the constituents’ particles.

**Figure 3:**
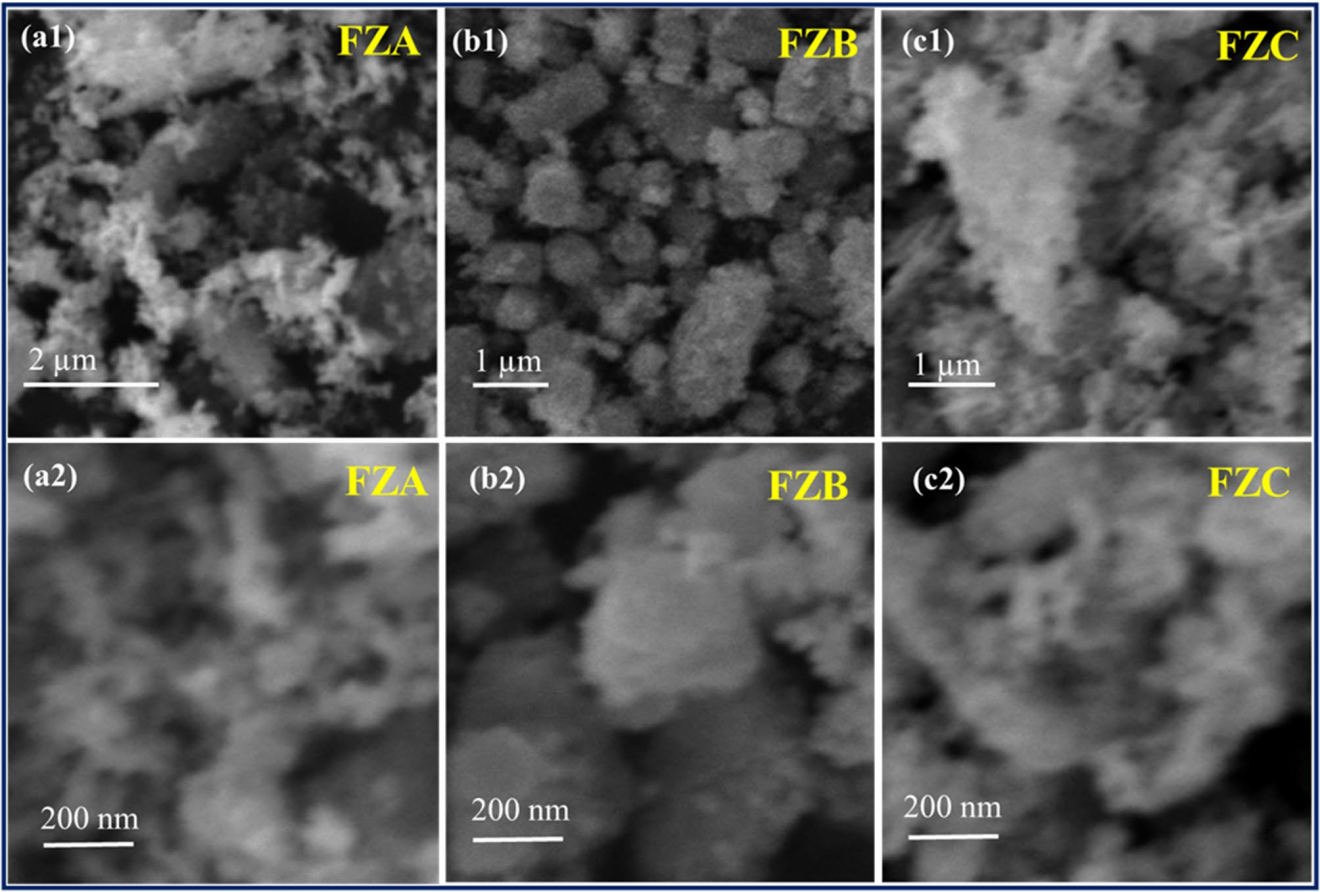
SEM images at low magnification (**a1**–**c1**), and (**a2**–**c2**) at high magnification for the samples FZA, FZB and FZC respectively.

Figure 3 shows the SEM micrographs of the FZNCs at low and high magnification to observe the morphology of the samples. Figure 3(a1–c1) depicts the SEM images at low magnification and (a2–c2) at high magnification for the MNC samples. From the SEM micrographs, we can clearly distinguish two differentiable sizes of the particles in all the FZNCs samples. The XRD results confirm that the crystallite size of the FZNCs lies in the range 40–50 nm and Fe_3_O_4_ nanoparticles taken as seeds are in the range around 10 nm. So, we can conclude from the micrographs that the large particle size depicted in the SEM micrographs are ZnO nanoparticles and smaller Fe_3_O_4_ nanoparticles are decorated on the surface of the ZnO. Figure 3(a1) and (a2) shows the SEM micrographs of the FZA samples at low and high magnification from which we can clearly observe that the small Fe_3_O_4_ particles are randomly dispersed in the samples. The ZnO and Fe_3_O_4_ particles are oriented erratically and thus the system will demonstrate isotropic behavior. This is well in agreement with the XRD results in which no separate peak is observed for the Fe_3_O_4_ nanoparticles which implies that the ZnO peaks suppress the Fe_3_O_4_ peak due to their much lower concentration. Whereas, for sample FZB, Fe_3_O_4_ nanoparticles were very well decorated at the surface of the ZnO particles as shown in Fig. 3(b1) and (b2). This makes the MNC sample highly uniform and isotropic as particles are uniformly distributed in the sample. The XRD results also confirm the same as the slight bump at the major peak of Fe_3_O_4_ is observed compared to the pure ZnO nanoparticles. Finally, Fig. 3(c1) and (c2) show the SEM micrograph for the sample FZC which contains the largest concentration of FN nanoparticles. Figure 3(c1 and c2) clearly illustrates that FN nanoparticles are agglomerated at the surface of the ZnO nanoparticles and they are appearing as a separate phase which can be corroborated from the XRD results where the Fe_3_O_4_ major peak is appearing abruptly.

**Figure 4:**
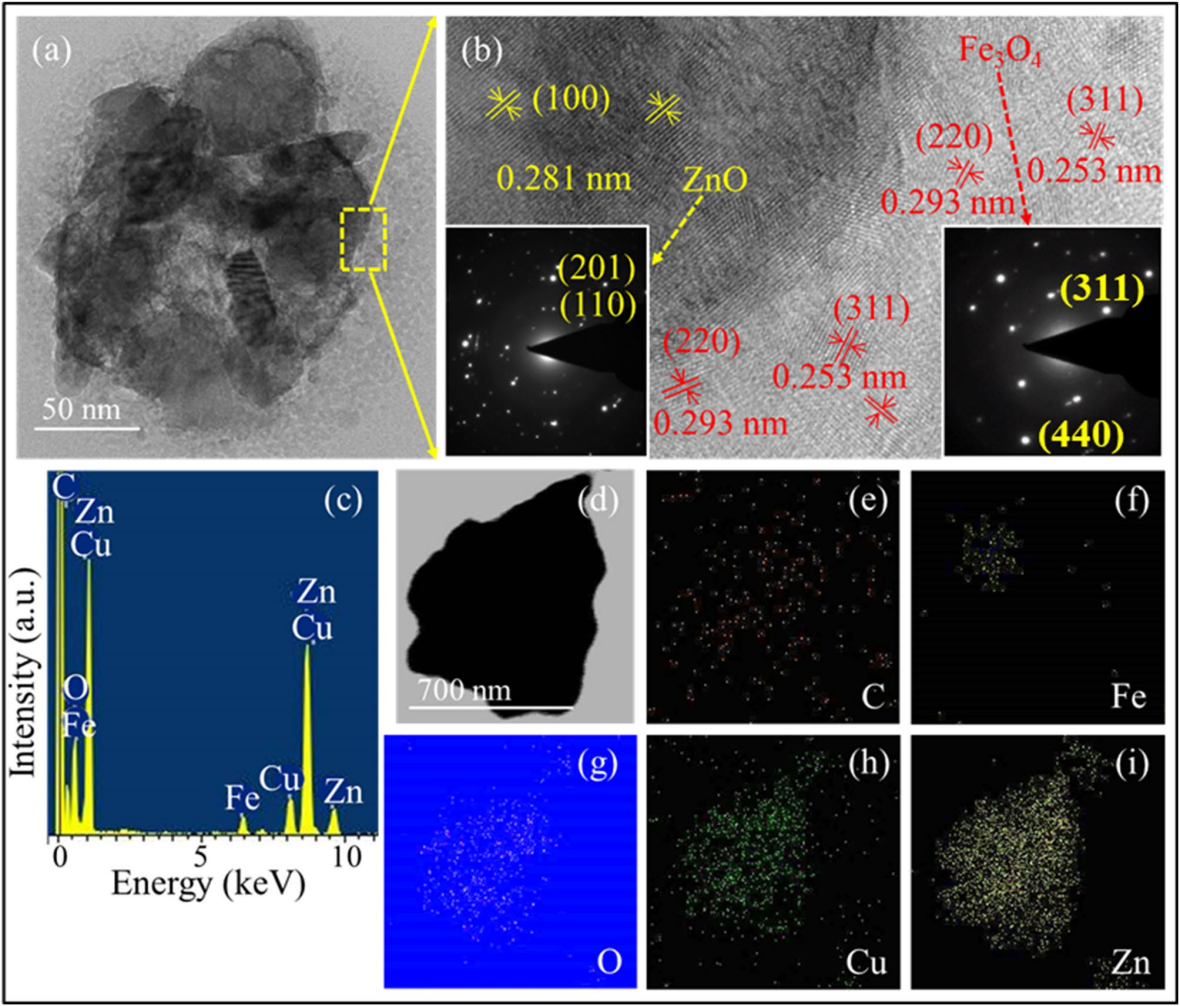
Electron micrographs and elemental mapping of sample FZB (**a**) bright-field image, (**b**) HRTEM micrograph, (**c**) EDS analysis, (**d**) area selected for elemental mapping (**e**–**i**) elemental mapping of individual elements (**e**) C, (**f**) Fe, (**g**) O, (**h**) Cu, and (**i**) Zn (the images were obtained using TEMCON software).

Figure 4(a) depicts the bright field image of FZB sample that clearly shows the presence of lower contrast small particles surrounding the dark core. The high-resolution transmission electron microscope (HRTEM) images and selected area electron diffraction (SAED) micrograph were taken at the interface to observe the present structures. The HRTEM image and SAED patterns shown in Fig. 4(b) confirm that the lower contrast small particles are Fe_3_O_4_ and dark big particles are ZnO. The existence of lattice fringes in both regions confirms the crystalline nature of the structures. SAED pattern present in the inset of Fig. 4(b) shows the polycrystalline nature of the nanocomposites. The uniform and well-organized lattice fringes of 0.281 nm corresponding to the (100) plane confirms the presence of hexagonal ZnO in the core. However, the lattice fringes of 0.253 and 0.296 nm corresponds to (311) and (220) planes, respectively, that affirms the presence of cubic crystalline phase Fe_3_O_4_^2,28,29^_._ The particle size of Fe_3_O_4_ is calculated to be range 2–10 nm. EDS analysis in Fig. 4(c) demonstrates various elements; Zn, Cu, Fe, and O present in the structure. Figure 4(e–i) shows the elemental mapping of C, Fe, O, Cu, and Zn elements, respectively obtained using TEMCON software. Figure 4 confirms the Fe_3_O_4_ surface decorated ZnO structure as shown schematically in Fig. 1.

**Figure 5:**
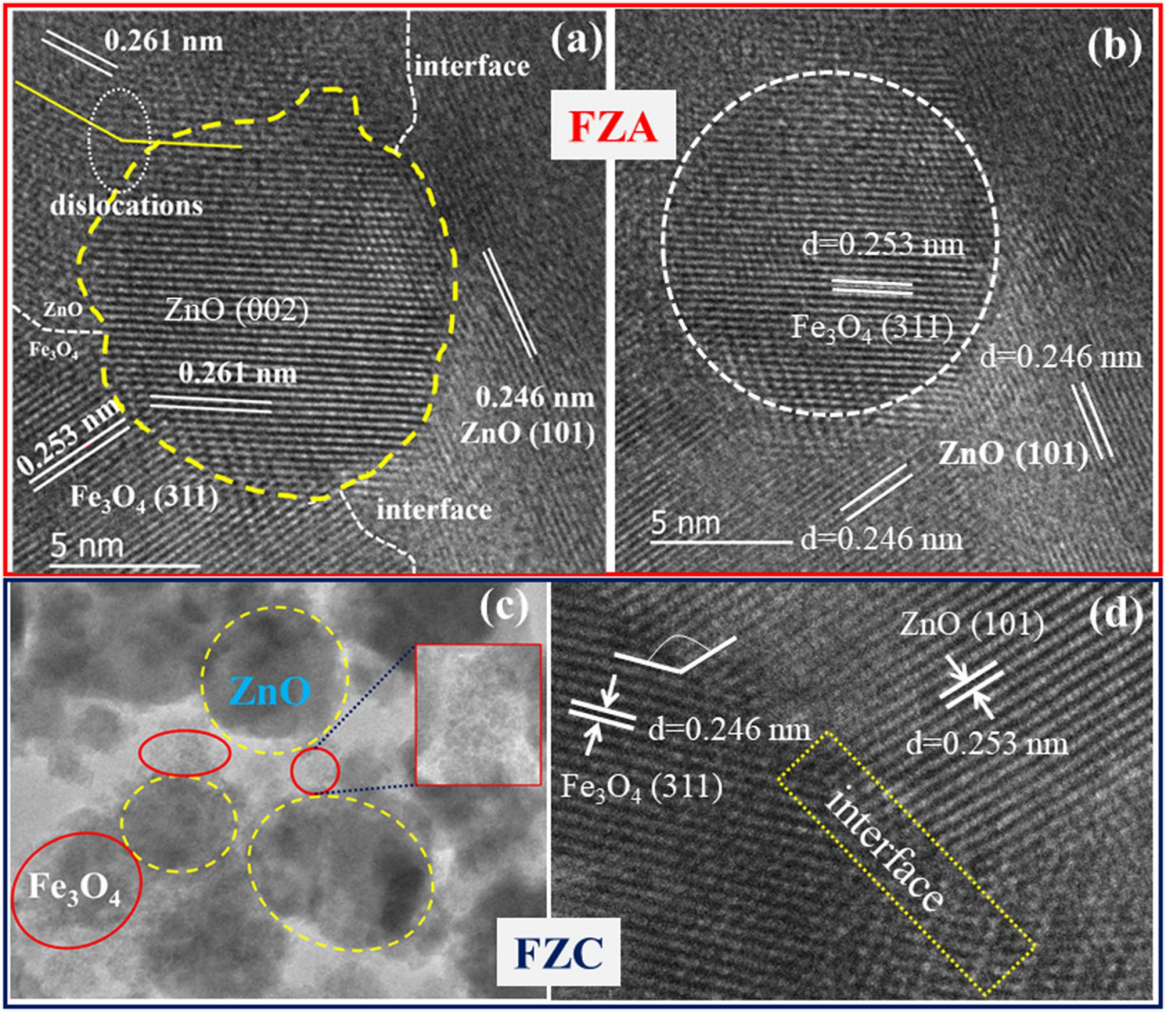
(**a**–**b**) HRTEM images of the sample FZA showing interfering grain boundaries of the ZnO and Fe_3_O_4_ nanoparticles. (**c**–**d**) HRTEM images of the sample FZC with overlapping grain boundaries.

Further, Fig. 5(a) reveals an HRTEM image of sample FZA which shows the presence of ZnO as well as Fe_3_O_4_ nanoparticles. Some nanocrystallites of hexagonal ZnO are distributed randomly with different interplanar spacings of 0.246 and 0.261 nm corresponding to (101) and (002) planes. Fe_3_O_4_ nanoparticles with an interplanar spacing of 0.253 nm correspond to (311) planes of cubic crystal structure were also noticed at some places^30,31^. Only a few numbers of Fe_3_O_4_ nanoparticles are observed due to less concentration taken while synthesizing these samples. Also, the dislocation in (002) planes of the ZnO is evident from Fig. 5(a) which is caused by the incorporation of the Fe_3_O_4_ (311) planes. The particle size of the Fe_3_O_4_ nanoparticles was found to be 11.2 nm (Fig. 5(b)). It is clear from Fig. 5(b) that Fe_3_O_4_ nanoparticles are decorated by ZnO nanoparticles. HRTEM images of sample FZC are shown in Fig. 5(c–d). Fe_3_O_4_ and ZnO nanoparticles were observed to be scattered, no uniform structure is present as depicted in Fig. 5(c). The excess amount of Fe_3_O_4_ is present as agglomerates around the ZnO nanoparticles which suggests that the formation of the composite is not suitable. An interface between these nanoparticles can be noticed in Fig. 5(d). Thus, the results are found in agreement with the proposed geometry that Fe_3_O_4_ and ZnO are present as nanocomposite structure with Fe_3_O_4_ nanoparticles are decorated by ZnO nanoparticles^32^.

So, from TEM results we can conclude that the sample FZB has a more uniform and suitable distribution of the magnetic nanoparticles over the surface of the ZnO. For low concentration (FZA) of the FN in the composite, the ZnO nanoparticles are not completely coated with Fe_3_O_4_ nanoparticles which produce anisotropic properties, while for the higher concentration (FZC) samples the nanocomposite formation is not suitable as both the nanoparticles are present separately. A small amount of Fe_3_O_4_ nanoparticles is present on the surface of ZnO and most of the parts are present as an agglomerate which hampers the properties of the nanocomposite.

**Figure 6:**
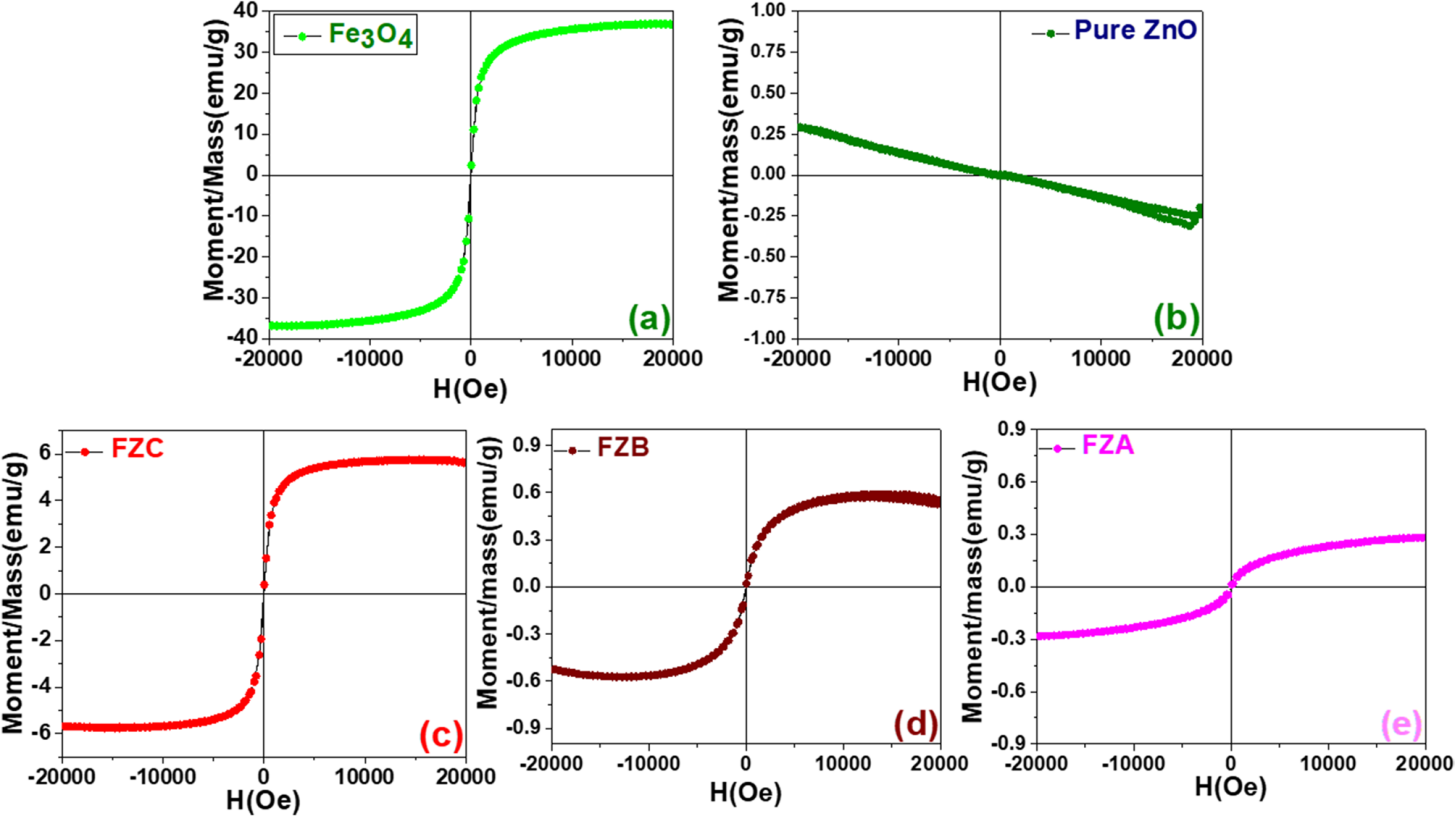
Room temperature dc magnetic measurement curves of (**a**) FN, (**b**) ZN, (**c**) FZC (**d**) FZB, (**e**) FZA samples.

The dependence on externally applied magnetic field of the static (dc) magnetization, m(H) for all the samples as recorded at room temperature in applied magnetic field up to 2 T is shown in Fig. 6(a–e), while M_s_ is summarized in Table 1. The M-H loop for the ZN shows diamagnetic behavior, which is well established in the literature and consistent with earlier reports. It is observed that the M-H loop of pure Fe_3_O_4_ shows SP behavior with Ms = 36.94 emu/g. Table 1 shows that FZA and FZB samples have low M_s_ = 0.2816 and 0.5565 emu/g, respectively, which can be well understood from the fact that these nanocomposite samples are of low FN concentration. Whereas, sample FZC shows higher Ms = 5.6976 emu/g as it has a higher concentration of FN loading. All the FZNC samples are showing perfect SP behaviors which reflects that these Fe_3_O_4_ seeds are present on the grain boundaries of the ZnO particles as a separate phase which is evident from the TEM micrographs. The composite behaves as SP and no staggering change in the M-H loop indicates that the Fe_3_O_4_ nanoparticles are uniformly mixed and forming efficient nanocomposite.

**Figure 7:**
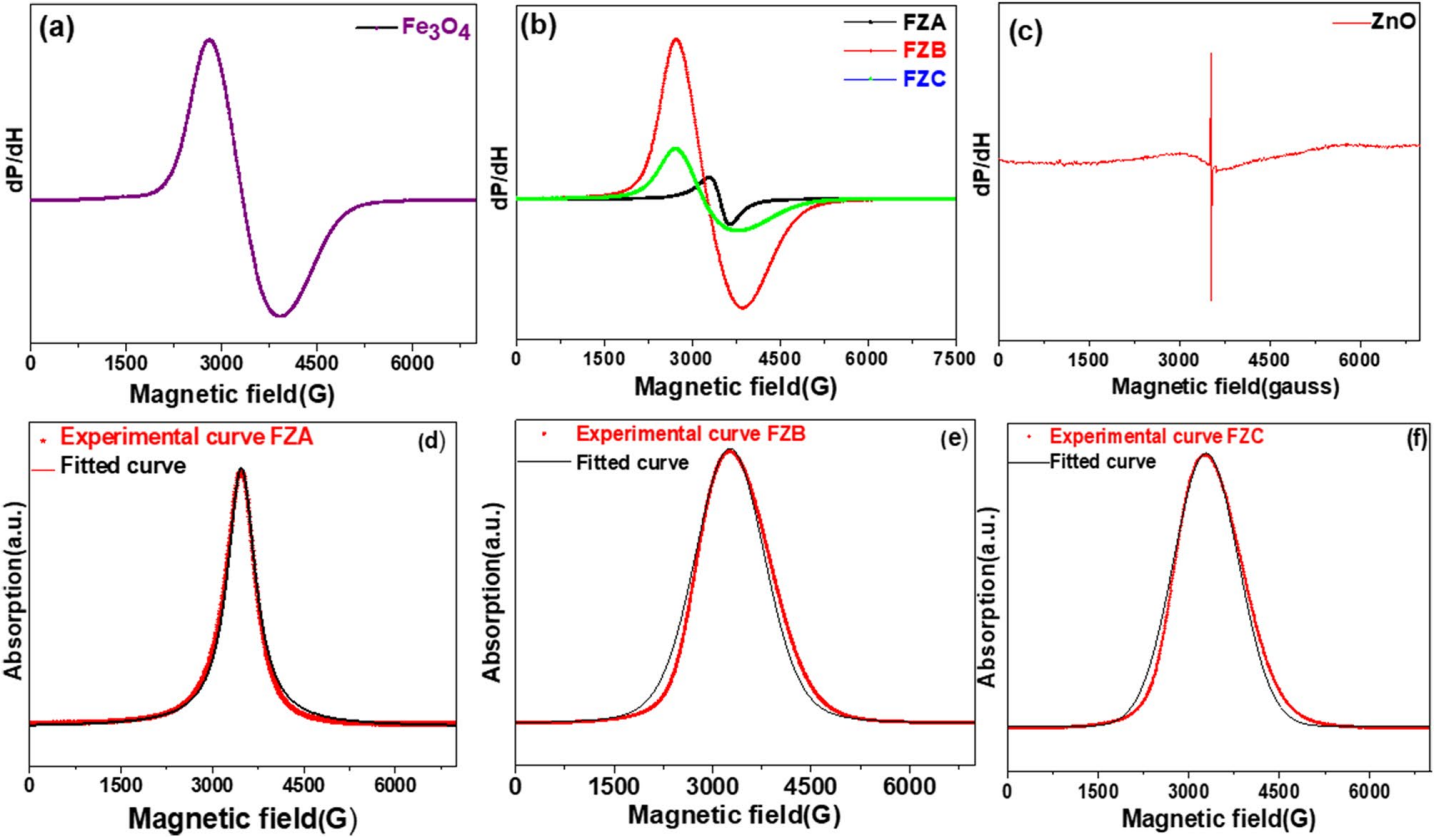
Room temperature FMR spectra of samples (**a**) FN, (**b**) FZNC, (**c**) ZN (**d**–**f**) fitted data of FZNC sample (**d**) FZA, (**e**) FZB, (**f**) FZC.

Furthermore, experimental and fitted FMR spectra of the samples have been shown in Fig. 7. All the FMR spectra were fitted using suitable distribution functions and spin resonance parameters were obtained from the best-fit curves. Figure 7 shows a single broad-spectrum for FZNC samples, implying that isolated Fe^3+^ and Zn^2+^ ions do not exist. The resonance line observed is the contribution from both Fe^3+^ and Zn^2+^ in a single phase^33^. FMR spectra of all the samples are symmetrical, but their resonance magnetic field ($H_{R}$) and peak-to-peak linewidth ($\Delta H_{PP}$*)* exhibit systematic variations. The resonance parameters such as *H*_R_, $\Delta H_{PP}$, *g*-factor, spin concentration (*N*s) and relaxation time (*T*_S_) are calculated for all the samples and listed in Table 1^34^. From Table 1 we can conclude that microwave resonance parameters strongly depend upon Fe_3_O_4_ concentration in the composite.

FMR spectra of both bare ZnO and Fe_3_O_4_ are in accordance with the earlier reported results in the literature^34,35^ and resonance in ZN and FN is observed at 3526 G and 3338 G, respectively^36^. Adding different amounts of Fe_3_O_4_ nanoparticles in the ZnO host matrix shifts the resonance spectra of ZnO in a systematic manner. Resonance is achieved at a lower value of the applied magnetic field with an increase in FN content in the composite. This results due to the increase in ferromagnetic interactions among Fe_3_O_4_ nanoparticles as the distance between them decreases on increasing of their proportion in the nanocomposite^37^. Another important parameter of the FMR spectrum is the *g*-value which provides the orbital contribution to the magnetic moment and is calculated using Eq. 1.

1 $$ g = \frac{h\nu }{{\mu_{B} H_{R} }} $$

Here, *h* is Planck’s constant, *ν* is microwave frequency, μ_B_ is Bohr’s magneton and H_R_ is the resonance magnetic field^37^. ZN and FN show signals at *g* = 1.969 and *g* = 2.0809, respectively, which is in accordance with the literature. The *g* value for Fe_3_O_4_ nanoparticles is greater than of bare ZnO nanoparticles, which is in line with the spin–orbit coupling. It can be elucidated from Eq. 2.

2 $$ g\sim2\left( {1 - \frac{\lambda }{\Delta }} \right) $$

Here, λ is the spin–orbit couplin*g* constant and ∆ is the energy level separation. λ takes negative values when the 3d shell is more than half full and is positive for half-filled 3d shells^38^. The *g*-value increased monotonically from 2.0005 to 2.17008 as the amount of Fe_3_O_4_ was stepped up in FZNC because of an increase in the composite, the increase in magnetic anisotropy possibly results in linewidth in the spin-orbital interactions. The large spin–orbit coupling can make FZNC a key material in future spintronic computing^23^. Furthermore, FMR spectra of ZN show extremely narrow linewidth which increases with an increase in Fe_3_O_4_ content and narrows down after reaching a maximum value at some optimized FN concentration. At lower concentrations of FN in the composite, the increase in magnetic anisotropy possibly results in linewidth broadening. Due to different directions of internal anisotropy fields of differently oriented facets of crystallites, the resonance curve is broadened since individual crystallites achieve resonance at their own magnetizing field. Another factor attributing to the broadening is the interference of crystalline fields of different crystallites in the region at the interface. As a result, both the zero-energy level splitting and the *g*-value of ferromagnetic ion located at or near the interface will get changed. There will be a random variation in the magnetic field required for resonance and the lines will get broadened^39^. Deepty et al.^40^ reported that enhancement in magneto-dipolar interactions partially leads to linewidth broadening. At higher concentrations, the phenomenon of exchange narrowing curtails the effectiveness of magneto dipolar interactions existing among the fine Fe_3_O_4_ clusters which result in the narrowing of linewidth^41,42^. This can be elucidated using the Eq. (3)^35,36^.

3 $$ \Delta H_{PP} \sim\frac{{H_{A}^{2} }}{{H_{ex} }}, $$

where *H*_A_ and *H*_ex_ are anisotropy and exchange fields, respectively^43^. The small value of linewidth at higher concentrations indicates that incorporation of Fe_3_O_4_ in ZnO reduces the energy loss and makes it suitable for high-frequency applications^44^. From the literature, it has been found that two-time effects or relaxation phenomena are involved in the resonance process i.e. spin–spin and spin–lattice relaxation^45^. The applied field *H* changes the direction of the field that each dipole experiences. As a result, the dipoles undergo a slight re-orientation and hereby relaxation occurs by small changes in the energy levels of the dipoles. Spin–spin relaxation time is a measure of the time required to establish thermal equilibrium in the spin system. It occurs when the applied field *H* is smaller than fluctuating internal fields *H*_i_ produced by the dipoles. The spin–spin relaxation time has been calculated from $\Delta H_{1/2}$ and *g*-value, listed in Table 1 using Eq. (4) ^40^:

4 $$ T_{s} = \frac{\hbar }{{g\mu_{{B{\raise0.7ex\hbox{${\Delta H_{1} }$} \!\mathord{\left/ {\vphantom {{\Delta H_{1} } 2}}\right.\kern-\nulldelimiterspace} \!\lower0.7ex\hbox{$2$}}}} }} $$

Sharp decrease in *T*_S_ value is observed with increasing Fe_3_O_4_ content after which it gets saturated showing no or little dependence on ferromagnetic particle concentration. This can be explained using the Heisenberg uncertainty principle^45^:

5 $$ \Delta E\sim\hbar \Delta \omega $$

At low Fe_3_O_4_ concentrations, due to spreading in the energy levels of dipole because of large dipole–dipole interactions, the dipoles own a wide range of frequencies. Consequently, a dipole will require a shorter time to reorient itself about a different precession axis^39^. At higher concentrations, competitive dipole–dipole and exchange interactions lead to the saturation in relaxation time values. Further, *N*s has been calculated using Eq. (6):

6 $$ N_{S} = \frac{9}{{4\pi^{2} }}\frac{{\Delta H_{{1{/}2}} }}{{g\mu_{B} }} $$

Here Δ*H*_1/2_ is the full width at half maximum of the absorption peak. It is seen from Table 1 that initially the spin number increases with increasing ferromagnetic particle concentration. After reaching an optimum concentration, it shows a very small incongruous decrease. The aberrant decrease in the value of *N*_S_ can be ascribed to a decrease in crystallization degree of the composite as the amount of Fe_3_O_4_ nanoparticles exceeds a critical concentration resulting in the decrease of the spin number of Fe^3+^^46^. Another effect of increasing the ferromagnetic particle concentration in the composite is that the line shape changes from Lorentzian to pseudo-Voigt, which is intermediate between Gaussian and Lorentzian and then to a Gaussian line shape. One of the parameters that is indicative of the line shape is the ratio of the full width at half-height, Δ*H*_1/2_ of the integrated spectrum to Δ*H*_PP_, Δ*H*_1/2_/Δ*H*_PP,_ is 1.657 for a Lorentzian, 1.135 for pseudo-Voigt and 1.191 for a Gaussian line shape. Broader wings of the Lorentzian peak shape as compared to Gaussian peak shape pinpoint to a large number of magnetic spins in sample FZA as compared to sample FZC. This is in conformity with our results for *N*s as shown in Table 1. Pseudo-Voigt line shape for sample FZC indicates an optimum balance between magneto-dipolar and exchange forces existing between magnetic moments. The spin resonance parameters suggest that the relationship of the concentration of the Fe_3_O_4_ and magnetic and spin dynamic properties are very complex^35,36^. Although, the magnetic saturation is quite straightforward which increases sharply with an increase in Fe_3_O_4_ concentration whereas the spin resonance parameters depend on the interaction among the constituents. FZB sample has a lower magnetic saturation value but has higher spin concentration resulting from the uniform distribution of the Fe_3_O_4_ nanoparticles which does not affect the magnetic ions distributions, while sample FZC which has high saturation, has low spin concentration due to spin canting effect. The present investigation allows FZNC to be used more effectively in various spintronic and optoelectronic devices.

## Conclusion

In conclusion, the room temperature spin dynamics in FZNC have been investigated in detail experimentally and the effect of Fe_3_O_4_ concentration on the properties of FZNC has been established. SP Fe_3_O_4_ nanoparticles were used as the seed to synthesize FZNC which are incorporated in the ZnO matrix occupying the grain boundaries. Fe_3_O_4_ and ZnO are present in the composite as a separate phase which converges that the composite will show properties of both FN and ZnO. The grain boundary incorporation of magnetic nanoparticles is achieved by producing the uniform radial magnetic field which allows the formation of chain structure while in-situ growth of the ZnO nanoparticles. The incorporation of FN particles on the grain boundaries has been confirmed through TEM and SEM. Further, HRTEM and SAED have been taken on both sides of the interface in the FZNC to confirm the constituents of the composite.

The static magnetization curve of FZNC shows an SP behavior which further complements the electron microscopy results that both FN and ZP are present as a separate phase and FN is not incorporated in the matrix of ZnO. Further, single broad FMR spectra of the composite show that no Fe and Zn ions are present in the samples, and large spin–orbit coupling is observed at higher FN loading. T_s_ of the samples also increases with an increase in FN loading and FMR spectra shape changes from Lorentzian to pseudo-Voigt. This is corroborated by the N_s_ results, which show large values of N_s_ for sample FZB rather than sample FZC due to balancing between magneto dipolar and exchange forces between magnetic moment. Large spin–orbit coupling in FZB samples can be achieved which is evident from the spin resonance parameters obtained for the samples. The present investigation depicts some unique and novel results as magnetic incorporation provides unique opportunity for the development of high-performance materials with the external control which unlocks the door for highly efficient devices.

## Experimental

To investigate the crystalline phase purity, X-ray diffraction (XRD) measurement of as prepared FZNC, Fe_3_O_4_ nanocrystals (FN), and ZnO particles (ZN) was performed using Rigaku Ultima –IV with Cu-Kα radiation (P = 3 kW, λ = 1.5406 Å). All the samples were scanned in the range 5° to 90° at 40 mV–40 mA at a step size 0.01 with a slow scan rate. Crystallite size and strain in the samples were calculated using the Williamson–Hall (W–H) method^47^. The morphology of the MNC samples was observed using SEM (Zeiss EVO MA-10 SEM) operating at 10 keV. Further, to analyze the nanocomposite formation and microstructural features, the TEM images at low and high resolution were obtained by JEOL JEM2100F operating at 200 kV. Also, the EDS spectrum and elemental mapping of the as-prepared nanocomposite samples were depicted using Jeol-JEM2100F standard attachment. The room temperature static dc magnetic measurement of the samples was performed using a vibrating sample magnetometer (VSM, Lake Shore 7304).

The microwave spin resonance investigation has been performed using Bruker EMX-10 spectrometer at room temperature using 100 kHz field modulation. The measurements were performed using X-band (9.85 GHz) microwave frequency, and a TM011 mode cavity. The distortion of the FMR spectra has been avoided by keeping the modulation amplitude less than or equal to one-third of the peak-to-peak linewidth. The FMR spectra were recorded at room temperature and various spin resonance parameters such as resonance field, Landé g-tensor, spin–spin relaxation time, and spin concentration were calculated. Further, these line spectra were fitted to the theoretical predictions to investigate the concentration dependence of the nanocomposite samples^33^. The instrument has been calibrated before each measurement by taking a dried DPPH (2,2-diphenyl-1-picrylhydrazyl) sample as standard, characterized by g-factor equal to 2.0036.
